# A diagnostic model for non-invasive urothelial cancer early detection based on methylation of urinary tumor DNA

**DOI:** 10.1186/s12935-025-03766-2

**Published:** 2025-04-15

**Authors:** Ningning Wu, Zhen Wu, Yanwen Wang, Anqi Zhang, Yongfei Peng, Yan Cheng, Hongsong Lei, Siwen Liu, Jie Zhao, Tianbao Li, Guangpeng Zhou

**Affiliations:** 1 BioChain (Beijing) Science & Technology Inc., Beijing, 102600 China; 2 Department of Urology, The First Affiliated Hospital of Dali University, 32 Carlsberg Avenue, Dali City, 671000 Yunnan Province China; 3 Central Laboratory, Liaocheng People’s Hospital, Liaocheng, 252000 Shandong Province China; 4 Department of Urology, The Second Affiliated Hospital of Kunming Medical University, 374 Yunnan-Burma Road, Wuhua District, Kunming, 650000 Yunnan Province China

**Keywords:** Urothelial cancer, UC diagnostic model, Methylation, Ut DNA, Non-invasive diagnosis

## Abstract

**Background:**

Diagnostic methods for urothelial cancer (UC) are often invasive, while urinary cytology, a non-invasive alternative, suffers from limited sensitivity. This study aimed to identify differentially methylated markers in urinary tumor DNA and develop a diagnostic method to enhance the sensitivity of non-invasive UC detection.

**Methods:**

Whole-genome bisulfite sequencing and deep methylation sequencing were employed to identify significantly hypermethylated UC-associated genes in clinical samples and public UC datasets. Further screening was conducted using tumor biopsies and urine samples from patients, leading to the selection of three hypermethylated UC markers. A diagnostic model based on these markers was constructed and validated in a cohort (N = 432) comprising patients with UC, other cancers, benign lesions, and non-UC urinary tract diseases.

**Results:**

Validation in a cohort of 432 subjects demonstrated that the UC diagnostic model, incorporating three hypermethylated markers (*VIM*, *TMEM220*, and *PPM1N*), achieved an overall sensitivity of 94.44% in 108 UC patients. Specificities were 96.34%, 90.76%, and 87.72% in 191 non-neoplastic individuals, 76 patients with benign lesions, and 57 patients with other cancers, respectively, resulting in an overall specificity of 93.52%. Methylation level analysis revealed significantly higher methylation (P < 0.001) for three markers in UC samples compared to non-UC samples. Furthermore, the model exhibited sensitivities of 80% and 88.57% for detecting stage 0a/0is and stage I UC, respectively.

**Conclusions:**

The UC diagnostic model demonstrates excellent diagnostic performance, particularly in the early detection of UC. This non-invasive approach, characterized by high sensitivity and specificity, holds significant potential for further clinical evaluation and development as a reliable tool for UC diagnosis using urine samples.

**Supplementary Information:**

The online version contains supplementary material available at 10.1186/s12935-025-03766-2.

## Background

Urothelial cancer (UC) is a common malignant neoplasm of the urinary system, ranking as the ninth most prevalent cancer worldwide [1, 2]. UC represents the predominant type of cancer in the bladder and urinary tract, in contrast to less common histological subtypes such as squamous cell carcinoma, sarcoma, lymphoma, and adenocarcinoma [3]. UC primarily includes upper tract urothelial carcinoma (UTUC) and urothelial bladder cancer (UBC) [4]. Among these, UBC is the most prevalent, accounting for approximately 80% of UC cases, with its incidence steadily increasing on an annual basis [5]. The initial symptoms of urothelial carcinoma are often subtle, leading to delayed diagnosis at middle or advanced stages, which significantly reduces the 5-year survival rate. For instance, in UBC cases, the 5-year survival rate exceeds 88% for non-muscle invasive bladder cancer (NMIBC), whereas it drops to 50% for muscle-invasive bladder cancer (MIBC) [6, 7]. Therefore, early screening and diagnosis of UC are critical for improving the 5-year survival outcomes of affected individuals.

Current diagnostic methods for UC include endoscopy, imaging techniques, and urine analysis [8]. While endoscopy and biopsy remain the gold standard for diagnosis, these procedures are invasive and often associated with limited patient compliance. Ultrasonography, a non-invasive and user-friendly imaging technique, has limited efficacy in detecting carcinoma in situ (0is) and small lesions [9]. Numerous studies have investigated urinary tumor markers for UBC, such as nuclear matrix protein 22 (*NMP22*) and bladder tumor antigen (BTA). However, their clinical utility is constrained by low sensitivity, which may result in missed diagnoses [10]. Consequently, there is an urgent need for a non-invasive and highly accurate diagnostic technique to detect UC at an early stage. Research on the methylation of exfoliated tumor cell DNA in urine has shown promise in addressing this challenge, particularly for detecting early-stage cancer lesions that are difficult to identify or unsuitable for endoscopic evaluation [11]. This approach not only enhances the sensitivity and specificity of UC detection but also gains increasing acceptance among patients [12].

DNA methylation is a critical epigenetic modification that regulates gene expression without altering the underlying genetic sequence [13]. Recent studies have highlighted its pivotal role in the initiation and progression of UC. Alterations in DNA methylation patterns are emerging as promising biomarkers for the early detection of UC. For example, Xiao et al. analyzed the specific DNA methylation profiles of urothelial bladder carcinoma and developed a method to detect these methylation characteristics in urine samples. Their findings demonstrated that changes in urine free DNA methylation patterns could serve as reliable biomarkers for the non-invasive detection, prognosis, and surveillance of UBC [14]. Similarly, Wang et al. assembled a cohort of 373 cases to develop UCseek, a highly sensitive model for detecting and monitoring UC progression. UCseek exhibited excellent performance when independently validated [15]. These findings underscore the potential of DNA methylation as a viable approach for addressing the challenges of early UC detection.

In this study, we performed differential methylation analysis using clinical samples and public datasets to identify and filter candidate genes. Further screening led to the selection of three hypermethylated markers. We also developed a method to detect these hypermethylated markers using quantitative methylation-specific PCR in clinical urine samples. Finally, we constructed a diagnostic model based on the three hypermethylated markers and validated it using clinical samples, including urine from 432 cases. This model has the potential to facilitate the early diagnosis of UC at a treatable stage.

## Methods

### Study design

**Fig. 1:**
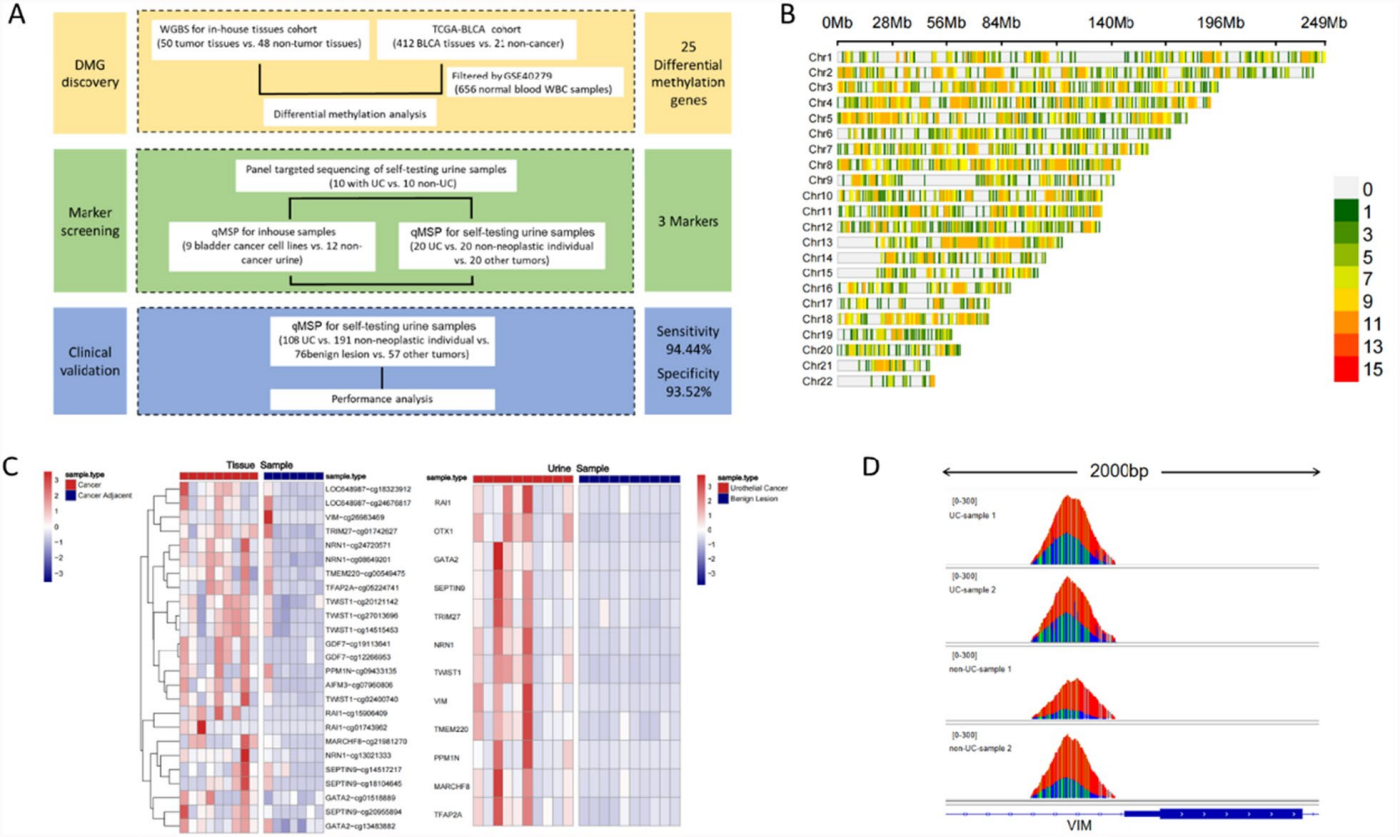
Research workflow and the preliminary screening of the methylation markers. **A** A workflow indicating the study design. DMG, differential methylation gene. WBC, white blood cell. WGBS, whole genome bisulfite sequencing. BLCA, bladder urothelial carcinoma. UC, urothelium carcinoma. qMSP, quantitative methylation-specific PCR. **B** The distribution of differential DNA methylation sites across autosomes analyzed by in-house WGBS data. The standard of filtering differential sites was defined as the absolute mean difference being greater than 0.05 (|delta|> 0.05) per 100 k genomic interval. The regions colored in green, less differential sites. The regions colored in red, more differential sites. The total number of differential sites on autosomes is 10,834. **C** Heatmaps showed methylation levels respectively for selected 25 potential markers and 12 potential markers in different groups of samples. Left panel, 9 UC tumor tissues and 7 non-UC tissues were tested by WGBS. Right panel, urine samples from 10 UC patients and 10 non-UC individuals were tested by panel-targeted deep methylation sequencing. The scale bars indicate the methylation levels scaled by Z-score, which red means high level and blue means low level. **D** The plots presented by IGV tool showed the *VIM* gene methylation in tissues and urine samples. The data processed were derived from BAM file alignments. Red represents T, thymine. Blue represents C, cytosine. Green represents G, guanine

This study was conducted in three distinct phases: the discovery stage of differentially methylated genes (DMG), the marker screening stage, and the clinical validation stage (Fig. 1A). In the DMG discovery stage, differential methylation analyses of an in-house whole genome bisulfite sequencing (WGBS) cohort and the TCGA-BLCA cohort were integrated to identify preliminary differential methylation sites for further selection. The second phase, the marker screening stage, focused on identifying specific markers for constructing the final diagnostic model. Urine samples collected in-house were utilized for panel-targeted sequencing and quantitative methylation-specific PCR (qMSP). The third phase, the clinical validation stage, involved incorporating the optimized markers into a diagnostic model and evaluating its performance in a clinical validation cohort.

### Study populations and samples

In the DMG discovery stage, a total of 98 tumor and non-tumor tissue samples were collected and analyzed from Liaocheng People’s Hospital. The in-house cohort comprised 9 bladder cancer tissues, 7 normal bladder tissues, 10 ovarian cancer tissues, 8 non-cancerous ovarian tissues, 8 prostate cancer tissues, 9 non-cancerous prostate tissues, 9 kidney cancer tissues, 9 non-cancerous tissues, 9 endometrial cancer tissues, 10 non-cancerous uterine tissues, 5 cervical cancer tissues, and 5 non-cancerous tissues. These samples were employed for whole genome bisulfite sequencing (WGBS), during which preliminary differential methylation sites indicative of pan-cancer hypermethylated genomic regions were identified. Additionally, differential methylation analysis was conducted using public datasets based on the 450 K array. The TCGA dataset included data from 412 cases of bladder urothelial carcinoma (BLCA) tissues and 21 matched adjacent non-tumor tissues. The GEO dataset (GSE40279) comprised data from blood leukocytes of 656 healthy individuals. The overlapping hypermethylated genes identified from these two datasets were carried forward for further analysis.

In the marker screening stage, urine samples were collected from 10 urothelial cancer patients and 10 healthy controls for targeted deep methylation sequencing, resulting in the identification of 12 selected markers. Subsequently, a quantitative methylation-specific PCR (qMSP) method was developed. Primers and probes for the 12 selected marker genes were designed, and qMSP was performed on 9 bladder cancer cell lines and 12 non-cancerous urine samples. The cell lines included M-UC- 3, SW780, J82, TCCSUP, SCaBER, HT- 1376, 5637, T24, and RT4. Six marker genes were chosen based on a cutoff threshold. Finally, three methylation markers were identified using logistic regression analysis on urine samples from 20 UC patients, 20 non-neoplastic individuals, and 20 patients with other tumors.

In the clinical validation stage, a three-marker diagnostic model was developed and its effectiveness verified using 432 urine samples, including 108 UC patients, 76 patients with benign diseases, 191 non-neoplastic individuals, and 57 patients with other cancers.

### Ethics approvals

Approval for this study was obtained from the Medical Ethics Committee of the participating hospital. All procedures adhered to the ethical standards of the responsible committee on human experimentation (institutional and national) and complied with the Helsinki Declaration of 1964 and its later amendments. Informed consent for participation in the study was obtained from all patients. The ethical approval number is as follows:

Liaocheng People’s Hospital, 2,023,151.

### Differential methylation genes (DMG) discovery

#### Bioinformatics analysis of public dataset identifying differential-methylation genomic sites

The methylation 450 K array data from 412 BLCA tissues and 21 paired adjacent normal tissues from TCGA were analyzed. Probes with a default value proportion exceeding 70% were excluded. For differential site selection, the R package ChAMP (version 2.21.1) [16] (450 k Chip Analysis Methylation Pipeline) was utilized, employing the BMIQ method for normalization and the Benjamini–Hochberg method to correct P-values (< 0.01). Selected sites were characterized by higher methylation in cancer tissues, with a mean difference > 0.2 (Tumor minus Normal > 0.2) between cancer and normal samples. Additionally, these sites were filtered against 656 blood leukocyte samples from healthy controls (GSE40279), retaining sites with a mean methylation level < 0.1 in blood leukocytes. This process led to the identification of the top differential-methylation genomic sites from TCGA-BLCA dataset.

#### Bioinformatics analysis of in-house WGBS dataset identifying differential-methylation genomic sites

The 30X WGBS was performed on 98 tissues (50 tumor tissues vs. 48 non-tumor tissues). The tissues included various cancer types and corresponding adjacent normal tissues, representing the significance of pan-cancer tissue methylation. DNA from paraffin-embedded tissues was extracted, fragmented to ~ 200 bp using ultrasonication, and subjected to bisulfite conversion. Library preparation was executed using the xGen™ Methyl-Seq DNA Library Prep Kit. Sequencing was conducted on the Illumina NovaSeq platform. The Bismark software (version 0.23.0) [17] was utilized to analyze WGBS data, applying a filtering criterion (Tumor minus Normal > 0.1), ultimately identifying top differential-methylation genomic sites from in-house WGBS dataset.

Then the top differential-methylation genomic sites from TCGA-BLCA dataset and in-house WGBS dataset were intersected to obtain the top 25 differential-methylation markers for subsequent selection.

### Candidate marker screening

#### Panel-targeted methylation sequencing

To further identify effective methylation markers, we constructed a 28.7 Mb panel based on differential methylation sites, encompassing 17,388 CpG islands and 3,311,582 CpGs. This panel facilitated deep methylation sequencing on 20 urine samples (10 UC vs. 10 non-UC) with an average depth exceeding 500X. DNA from urinary exfoliated cells was extracted, fragmented to ~ 200 bp, bisulfite-treated, and prepared for library construction. A custom TWIST probe panel was employed for hybridization and capture, followed by sequencing. Data analysis using Bismark software (version 0.23.0), with a criterion (Tumor minus Normal > 0.1), identified 12 differentially methylated genes.

#### The details of the in-house panel are as follows:

Targeted bisulfite sequencing was performed for all cfDNA and gDNA samples. The size of interested region is 28.7 Mb, covering 17,388 CpG islands (CGI) and 3,311,582 CpGs. Genes related with cancer and methylation were obtained from multiple databases, including 976 cancer driver genes, 1,220 tumor suppressor genes, 1,284 methylation related genes across multiple cancers, 732 cancer associated genes from COSMIC (Catalogue Of Somatic Mutations In Cancer, https://cancer.sanger.ac.uk/cosmic) and 26 leukemia genes. First, the promoters located 2.5 kb upstream and 500 bp downstream of the transcription start sites (TSS) of the genes were merged to form our region of interest. Second, CGI regions within these genes, as well as CGI-shore and CGI-shelf regions, were incorporated into the region of interest, which may be associated with cancer dysfunction. Third, a comparative analysis identified the 1,000 most significantly differentially methylated positions (DMPs) relative to normal tissues for various cancer types from TCGA databases, including LUAD, LUSC, COAD, LAML, BRCA, CESC, ESCA, LIHC, OV, PAAD, PRAD, STAD, and BLCA.

#### Quantitative methylation-specific PCR (qMSP)

The qMSP procedure was conducted as described in the study published by Nie et al. [18]. After bisulfite conversion of the extracted DNA, it served as a template for PCR amplification in a total volume of 30 µL, comprising 15 µL of prepared reaction mix and 15 µL of bisulfite-treated DNA. The PCR amplification program consisted of: 94 °C for 20 min; (93 °C for 30 s; 57 °C for 35 s) for 45 cycles; and 40 °C for 5 s. The PCR instruments employed were the Applied Biosystems 7500 Fast Real-Time PCR System (Applied Biosystems) or the SLAN- 96S Fully Automated Medical PCR Analysis System (Shanghai Hongshi Medical Technology Co., Ltd.). Each experiment included patient-extracted samples, positive controls, and negative controls.

Primers and probes were designed for the selected 12 differentially methylated genes. Then qMSP was performed using DNA extracted from 9 bladder cancer cell lines and 12 non-cancerous urine samples. Subsequently, six candidate methylated DNA markers were identified, exhibiting a sensitivity greater than 60% in bladder cancer cell lines and a specificity exceeding 80% in non-cancerous urine samples. The qMSP was then conducted on samples from 20 UC patients, 20 non-neoplastic individuals, and 20 patients with other cancers. The cut-off value for Ct was set at 45, with positive cases defined as Ct < 45. The three differential methylation genes with the highest accuracies were selected.

#### Urine sample processing

Urine samples (40–50 mL) were collected using Urine DNA Storage Tubes containing a sample-protection solution (CWBIO Co., Ltd.) prior to patients undergoing surgery and therapy. The urine was transferred into a 50 mL centrifuge tube and centrifuged at 4,000 rpm to retain the precipitate. The precipitate was washed with PBS and stored at − 80 °C.

### The diagnostic model construction and verification using clinical samples (432 cases)

A three-marker diagnostic model was constructed based on the principle that a single positive result indicates a positive diagnosis for UC, while three negative results indicate a negative diagnosis. This logic was supported by ROC curve analysis, which demonstrated its superiority over logistic regression. The three genes selected were the top-accuracy genes among the six candidate methylated DNA markers. To verify the performance of the model, qMSP for the three genes was conducted on urine samples from 108 UC patients, 191 non-neoplastic individuals, 76 patients with benign lesions, and 57 patients with other tumors, applying the diagnostic logic of the model.

## Results

### Discovery of potential DNA methylation markers for urothelial cancer

To identify potentially reliable candidate biomarkers for UC, the current study followed three main steps, including discovery of differentially methylated genes (DMGs) using public data in conjunction with our own cohort, followed by marker screening conducted on multiple types of samples, and finally, the clinical validation for diagnostic model in urine samples (Fig. 1A). In the DMG discovery step, we utilized publicly available 450 k array data from the TCGA database (TCGA-BLCA), including data from 412 bladder cancer samples and 21 paired adjacent non-cancerous tissues. Differential methylation analysis was employed to identify genomic regions with significantly higher methylation levels in UC tumor tissues compared to adjacent non-tumor tissues. To ensure the accuracy of our differential methylation analysis and avoid false-positive results, we excluded interference from leukocyte-derived DNA, which could obscure distinctions between malignant and benign diseases of the urinary tract. Since our proposed non-invasive diagnostic method relies on cell-free DNA (cfDNA) extracted from urine, it was critical to eliminate the influence of leukocyte DNA methylation. To achieve this, we utilized publicly available genome-wide methylation data from blood leukocytes of 656 healthy individuals (GSE40279) as a filter. Specifically, we excluded loci with an average methylation level greater than 0.1 in leukocyte DNA, ensuring that leukocyte-derived genomic DNA in urine would not affect the detection results. We prioritized the top 100 candidate markers for downstream research and displayed the differential methylation levels between 412 bladder cancer samples and 21 paired adjacent non-cancerous tissues from TCGA in Supplemental Fig. 1.

To further narrow the range of candidate markers, we obtained 98 clinical tissue samples (50 tumor tissues and 48 non-tumor tissues) from the hospital specified in the Methods section for 30X WGBS analysis. Methylation levels were calculated across 100 kb genomic intervals, which identified significantly differentially methylated sites. The distribution of these sites across autosomes is presented in Fig. 1B. Based on the mean difference in methylation levels between groups, 25 differentially methylated sites were selected from the 100 candidate markers described earlier. These sites were further examined in 9 UC tumor samples and 7 non-tumor tissues from the 98 in-house clinical tissue samples (Fig. 1C, left panel**)**.

To further evaluate whether the candidate markers identified in comparisons between cancerous and non-cancerous tissues could similarly distinguish urine samples from cancer patients and non-cancer patients, we collected urine samples from an additional 10 UC patients and 10 patients with benign diseases. Using an in-house panel (28.7 Mb, details provided in the Methods section), targeted deep methylation sequencing (630 M reads) was performed on these 20 urine samples. Differential analysis identified 12 differentially methylated genes from the 25 previously selected sites, with their methylation levels visualized in a heatmap (Fig. 1C, right panel). Among these 12 hypermethylated candidates, *VIM* was previously reported to be associated with urothelial cancer [19], prompting us to focus on this gene. Differences in methylation levels detected in both tissue and urine samples are shown in Fig. 1D.

### Further marker screening and construction of a non-invasive diagnostic model for urothelial cancer

**Fig. 2:**
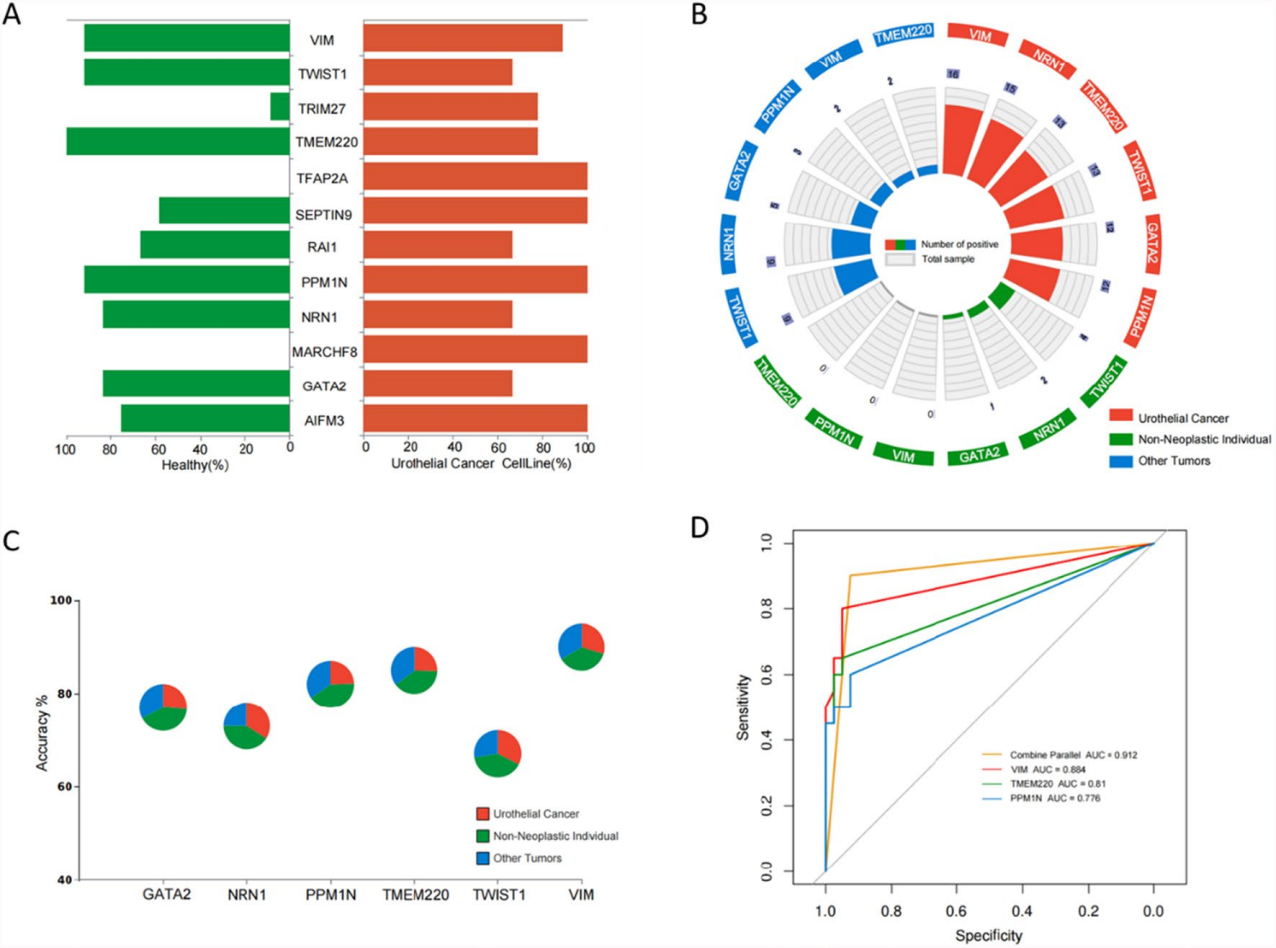
Construction of three-marker diagnostic model. **A** The sensitivity and specificity of selected 12 candidate markers in 9 UC cell lines and 12 healthy people urine samples. Testing method, qMSP. **B** The circle bar plot showing the respective positive cases of 6 candidate markers in UC patients (N = 20), non-neoplastic individuals (N = 20) and other tumor patients (N = 20). Testing method, qMSP. **C** The pie chart showing the accuracy of 6 candidate markers (corresponding y-axis value of each pie). The components of each pie represent the numbers of true positive case in UC group (red), true negative case in non-neoplastic group (blue) and true negative case in other tumors group (purple). The x-axis represents gene names of 6 candidate markers. **D** The ROC curves of four diagnostic markers, accuracy top 3 genes (*VIM*, *TMEM220* and *PPM1 N*) and Combine Parallel (three-marker diagnostic model), shown in the graph for distinguishing 20 UC patients from other 40 non-UC cases. The AUC values for each diagnostic markers were marked in the graph legend. Three-marker diagnostic model was based on the logic of single-gene positive indicating positive and triple negative indicating negative

To identify the most effective markers for UC detection among the 12 candidate DMGs, we evaluated their methylation levels in 9 UC cell lines and 12 urine samples from healthy volunteers. Quantitative methylation-specific PCR (qMSP) assays were developed for each candidate marker, resulting in average detection rates exceeding 60% for all 12 genes across the cell lines (Fig. 2A). Six of these candidate DMGs demonstrated sensitivity > 60% and specificity > 80% and were selected for further evaluation as potential UC biomarkers.

In the final step of the iterative screening process, 60 urine samples were analyzed, including samples from 20 patients with urothelial cancer, 20 non-neoplastic individuals with other urinary tract-associated disorders, and 20 patients with other cancers. qMSP detection of the six candidate markers was performed on these 60 urine samples using the same cutoff value and criteria (CT < 45, positive; CT ≥ 45, negative). Among these DMGs, *VIM* exhibited the highest detection rate (80%) in UC patients (Fig. 2B). Further analysis of marker accuracy revealed that *VIM*, *TMEM220*, and *PPM1 N* demonstrated the highest accuracy among the 60 urine samples (Fig. 2C).

Based on these findings, a diagnostic model was constructed using these three markers, *VIM*, *TMEM220*, and *PPM1 N*. The diagnostic model was designed such that a single positive result for any of the three markers (*VIM*, *TMEM220*, or *PPM1 N*) indicated the presence of UC, while all three negative results indicated the absence of UC. Receiver operating characteristic (ROC) analysis revealed an area under the curve (AUC) of 0.912 for the three-marker combined model, which was higher than the AUC values obtained for any single marker (AUC range: 0.776–0.884; Fig. 2D). These results collectively suggest that this three-marker combined model provides sufficient accuracy and sensitivity for the non-invasive diagnosis of UC in urine samples, making it a promising tool for clinical application.

### Validation of the three-marker UC diagnostic model in a clinical validation cohort

**Fig. 3:**
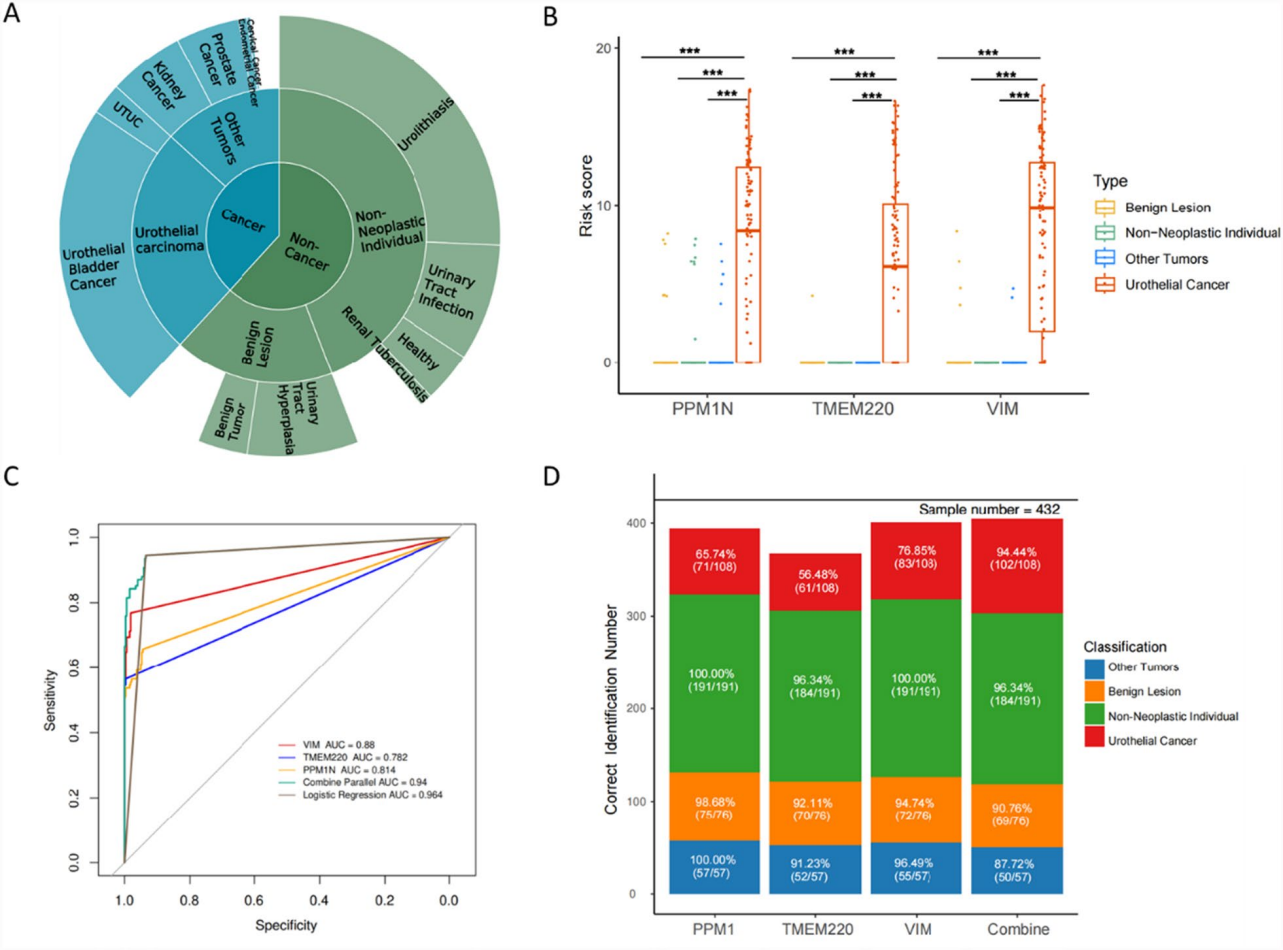
Performance verification of the three-marker diagnostic model in the model-testing cohort. **A** The three-layer pie chart showing the detail types of cases included in the model-testing cohort (N = 432). **B** Scatter boxplot showing the risk score of qMSP for the three methylation markers included in the model. Risk Score equals to delta-CT value (45-CT). *** means P value < 0.001. **C** The ROC curves of VIM, TMEM220, PPM1 N, Combine Parallel (three-marker diagnostic model) and logistic regression model in distinguishing the UC cases from all the non-UC cases in model-testing cohort. The AUC values for the different categories were marked in the graph legend. Three-marker diagnostic model was based on the logic of single-gene positive indicating positive and triple negative indicating negative. Logistic regression model was based on the logistic regression algorithm of VIM, TMEM220 and PPM1 N. **D** Vertical stacked bar plots of correct identifications as UC-positive or UC-negative, including true positive numbers and percentages in UC cases, as well as true negative numbers and percentages in benign lesion cases, non-neoplastic cases, and other tumor types

To validate the accuracy and sensitivity of the three-marker diagnostic model, we analyzed 432 urine samples, including 108 urothelial carcinoma (UC) cases, 191 non-neoplastic urinary tract disorder cases, 76 benign disease cases, and 57 cases with other cancers. Table 1 provides cohort statistics and patient demographic information. To assess the model's specificity in distinguishing UC from other malignancies, the cohort included samples from patients with renal cancer, prostate cancer, endometrial cancer, cervical cancer, and other cancers (Fig. 3A). Risk analysis demonstrated that all three genes had significantly higher methylation scores in UC samples compared to the other three groups (P < 0.001), further confirming the elevated methylation levels of these genes in UC patients relative to non-UC individuals (Fig. 3B). Additionally, the three-marker diagnostic model achieved an area under the curve (AUC) value of 0.94 for detecting UC patients in the full validation cohort (n = 432), outperforming single-gene detection, which yielded AUC values ranging from 0.782 to 0.88 (Fig. 3C). Parallel analyses for distinguishing UC patients from various other groups in the cohort are presented as ROC curves in Supplemental Fig. 2A–2D, with AUC values for the three-marker diagnostic model ranging from 0.911 to 0.954. An analysis of sensitivity for UC detection and specificity among other cancers, benign lesions, and non-neoplastic individuals was conducted for the three-marker combined model (*PPM1 N*, *TMEM220*, and *VIM*) as well as for each marker individually. Among the individual markers, *VIM* demonstrated the highest accuracy, correctly identifying 92.82% of the samples (401 out of 432). However, *VIM* exhibited lower sensitivity (76.85%) compared to the three-marker combined model, which achieved a sensitivity of 94.44% (Fig. 3D). Based on these findings, the three-marker diagnostic model demonstrated superior accuracy and sensitivity, making it a robust tool for distinguishing UC patients from non-UC individuals.

**Table 1:**
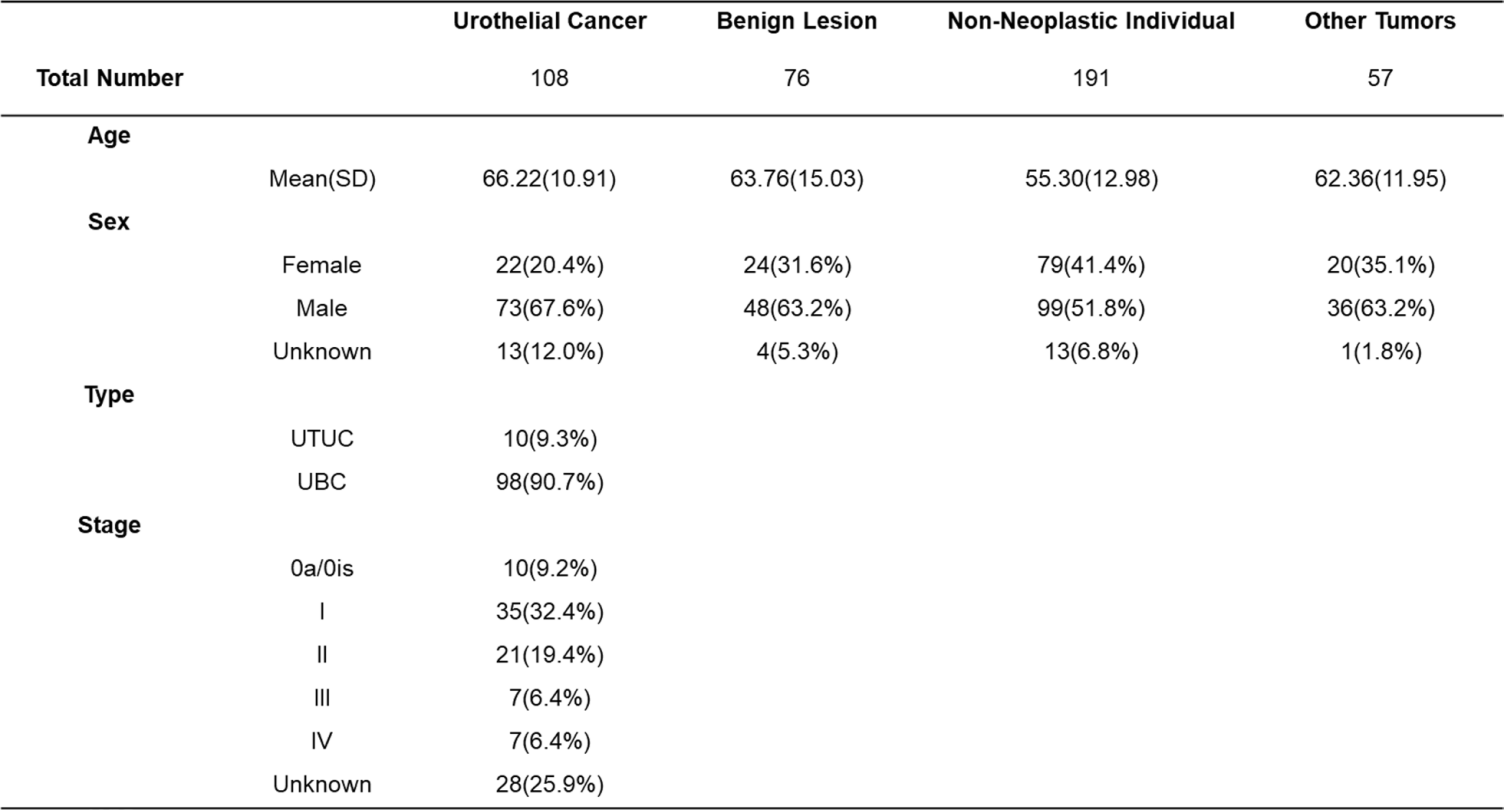
The clinical information of the model-testing cohort

Model-testing cohort, 432 cases in total, containing 108 UC patients, 191 non-neoplastic individuals, 76 benign lesions patients, and 57 other cancers patients.

**Fig. 4:**
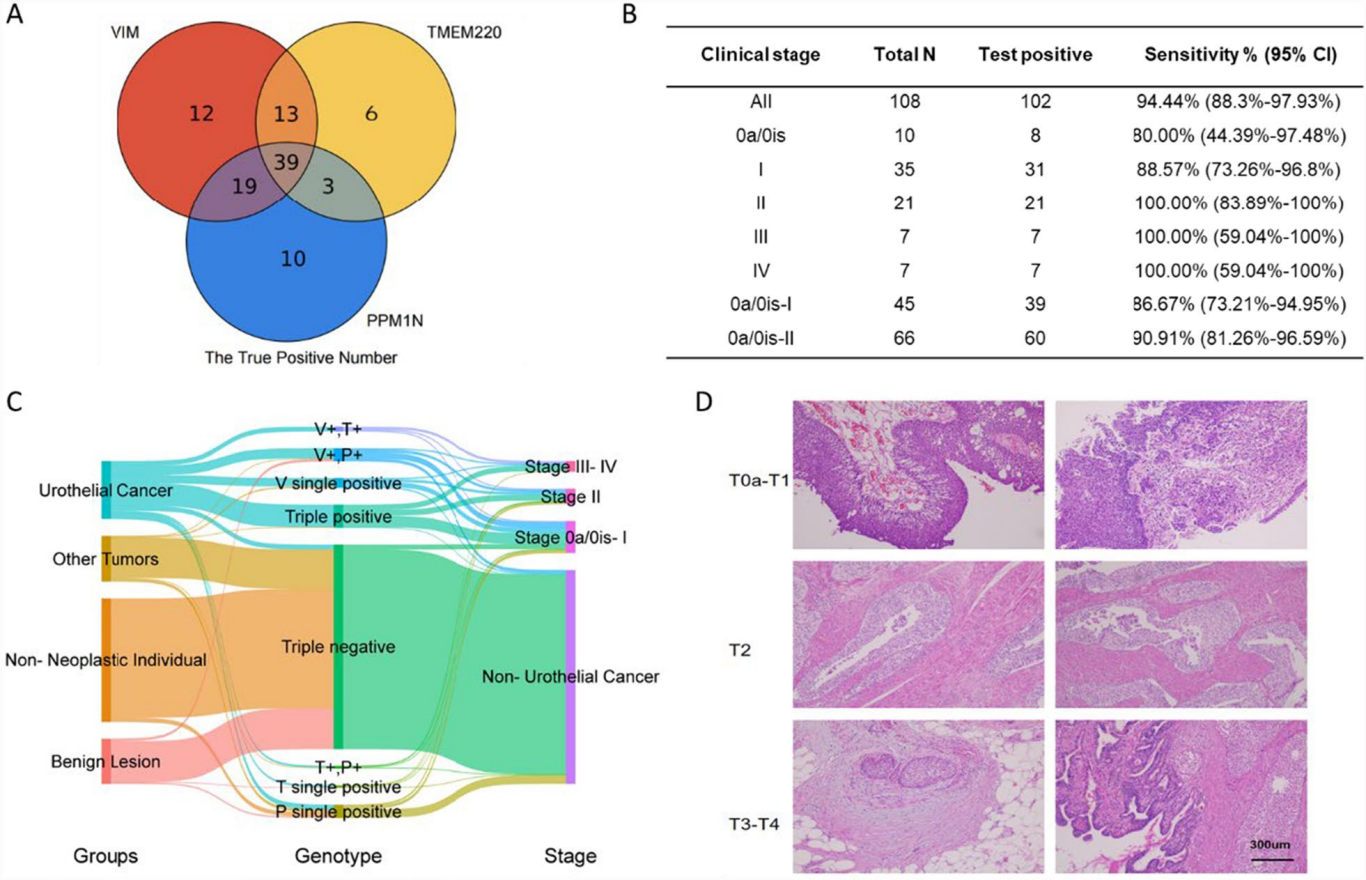
The application of diagnostic model in different urothelial cancer stages. **A** The correctly detected numbers (true positive numbers) of three single-gene diagnostic markers and their overlaps. **B** The true positive number and the sensitivity of three-marker diagnostic model in different clinical stages of the 108 UC patients in model-testing cohort. **C** Sankey diagram showing the component and the grouping trend of different ways of classification. The cases included all urine samples used in the study, except for the 30 cases lacking definite staging information. V represents for VIM. T represents for TMEM220. P represents for PPM1 N. **D** HE-staining pictures of tumor tissues sections. Histological photos of 6 representative cases were presented, 2 cases of stage 0a – stage I, 2 cases of stage II, and 2 cases of stage III – stage IV. Scale bar represents for 300 μm

Further analysis of different marker combinations revealed that *VIM* alone could detect the majority of true positive samples (83/102), but its performance was significantly improved when combined with *TMEM220* and *PPM1 N* (Fig. 4A). This finding highlights the higher detection rates achieved by the three-marker diagnostic model compared to each individual marker. To evaluate the effectiveness of the three-marker diagnostic model in detecting early-stage UC, we compared its sensitivity across samples from different UC stages. The model demonstrated a sensitivity of 80.00% (95% CI, 44.39%–97.48%) for stage 0a/0is samples, 88.57% (95% CI, 73.26%–96.8%) for stage I samples, and 100% (95% CI, 83.89%–100%) for stage II samples (Fig. 4B). Stratification of patients by disease group, genotype, and cancer stage further revealed that the overwhelming majority (97.70%) of triple-negative cases were non-urothelial cancer individuals, with only 2.30% of triple-negative cases corresponding to stage 0a/0is–I UC. Conversely, nearly all triple-positive cases (38/39) were stage 0a/0is–IV UC cases, with the only non-UC triple-positive case belonging to the other cancers group. These findings suggest that patients positive for hypermethylation of all three markers are highly likely to have malignant lesions, even if not UC, based on clinical diagnosis (Fig. 4C). Additionally, a radar chart summarizing the performance indicators of the three-marker diagnostic model in the model-testing cohort (n = 432) showed a positive predictive value (PPV) of 82.93% and a negative predictive value (NPV) of 98.06% (Supplemental Fig. 3). Histological analysis using H&E staining of representative tumor tissue sections from cases that tested positive in the model-based detection showcased samples from different UC stages, including stage 0a/I, II, and III/IV (Fig. 4D).

## Discussion

In the field of early UC diagnosis, there is a need for noninvasive methods with excellent performance. Our study developed a diagnostic approach for detecting urothelial cancer by assessing the DNA methylation levels of three markers (*VIM*, *TMEM220*, and *PPM1 N*) in urine. During the process of marker screening, our methodology leverages whole genome bisulfite sequencing (WGBS), targeted panel methylation sequencing, and qMSP to identify and validate stable hypermethylation events across multiple sample types, including tissues, urine, and urothelial cancer (UC) cell lines. After confirming the final selection of three marker genes, a diagnostic model was established based on qMSP for the three markers. Finally, we verified the diagnostic performance of the model in a testing cohort including 432 individuals.

A key novelty of this work lies in our integrated approach to marker screening, which not only uncovers differentially methylated sites but also provides insights into the genomic context of these alterations. While the overall methylation levels of the genome in tumors are generally lower than those in normal tissues, which can lead to decreased genome stability and increased mutation rate [20], there are still many silenced tumor suppressor genes with hypermethylation in tumor tissues being identified [21]. Studies in oncology have shown that promoter CpG island hypermethylation is frequently linked to the repression of tumor suppressor genes and can serve as a robust diagnostic indicator [22, 23]. Therefore, to facilitate the construction of a qMSP-based detection approach, we chose genes that are highly methylated in tumor tissues as candidate markers.

Our analyses suggest that the three markers, *VIM*, *TMEM220*, and *PPM1 N*, exhibit significantly higher methylation levels in urinary tumor DNA (utDNA) relative to non-cancer specimens. Further inquiring their genomic regions, it finds out that these markers’ hypermethylation events occur within promoter CpG islands, which could infer the marker gene silencing and the association with tumor biology. Then we further considered the biological relevance between marker methylation and their diagnostic utility.

*VIM* presents a cytoskeletal protein, Vimentin, which functions in maintaining cell structure and plays a crucial role in cell mechanosensing and signaling transduction. The *VIM* knockout mice showed that the recovery of tissues following various types of injuries was altered [24]. Besides, several studies reported that *VIM* is high methylated in cancer tissues or peripheral blood and its aberrant methylation will cause cancer cells more aggressive and metastatic, as well as proposing that *VIM* could be a diagnostic biomarker of cervical cancer, colorectal cancer and gastric cancer [25–27]. It can be speculated that hypermethylation events occurring in the promotor of *VIM* gene suppressed its expression in carcinoma, leading to the promotion of the mesenchymal-epithelial transition process [28], whose abnormal regulation can be considered to facilitate tumorigenesis and progression.

*TMEM220* is the Transmembrane Protein 220, a component of membrane and signaling transduction molecule. Previous studies indicated that *TMEM220* is hypermethylated in gastric cancer and colon adenocarcinoma [29, 30]. Other investigators reported that the expression level of *TMEM220* is low in hepatocellular carcinoma and is associated with poor prognosis in patients, indicating that downregulation of *TMEM220* promotes the progression of hepatocellular carcinoma [31]. Considering that the highly methylated DNA region we used for marker detection is also located in the promoter region of *TMEM220*, the hypermethylation of the marker suggests its downregulation of mRNA expression levels in urothelial cancer tissues. According to Li et al., aberrant expression of *TMEM220* would disturb the β-catenin signal and *FOXO3* subcellular localization, resulting in abnormal downstream gene expression [31]. Thus, alterations in *TMEM220* promoter methylation in UC may contribute to the similar pathway variations and promote cancer progression.

*PPM1 N* is Protein Phosphatase, Mg^2+^/Mn^2+^ dependent 1 N, a member of Metal-dependent Ser/Thr protein phosphatase PPM family, which was predicted to be involved in the regulation of canonical Wnt signaling pathway [32, 33]. Although we detected the hypermethylation of promotor region in *PPM1 N* gene, the biological relevance about *PPM1 N* methylation is less studies. Thus, our data are the first to report its significant hypermethylation in utDNA from UC patients, suggesting a potential role in tumor progression.

The alert for urothelial cancer in early stage is important to therapy and outcomes. The current primary diagnostic methods include cystoscopy and cytology [3]. The cystoscopy is invasive and often associated with discomfort, while the non-invasive cytology showing a poor sensitivity (25%− 35%) [34–36]. Our noninvasive diagnostic model based on qMSP demonstrated an overall sensitivity of 94.44% and specificity of 93.52%, with especially high performance in detecting early-stage (stage 0a/0is sensitivity: 80.00%) UC. Furtherly, in the non-invasive methods of early screen of UC, detection in urine has more advantages than detection in blood samples. Besides the easy accessibility, urine-based testing allows for more timely detection of bladder cancer lesions compared to blood-based testing. Because the exfoliation of malignant cells and the tumor DNA release from the lesions on urinary tract always happens before the invasion of them, including migration to adjacent tissue and vascular invasion [37, 38]. Moreover, comparing with detecting ctDNA of colon cancer et al., which the circulating tumor DNA from adenocarcinoma may need to pass through the vascular wall, the urinary tumor DNA (utDNA) could be detected in urine directly after releasing from malignant epithelium [39]. This phenomenon likely contributes to the high early-detection performance of the biomarker model, enhancing its diagnostic utility. Besides, the detection technology we employed is quantitative methylation-specific PCR, which is economical and feasible. These indicate our findings could be a potential early diagnosis product with remarkable effectiveness.

The diagnostic model indicates this convenient detection method could improve early detection and patient compliance. Importantly, comparing to previously published diagnostic model for bladder cancer which exhibited an overall sensitivity of 90.0% and a specificity greater than 80% [40], our model displayed a more satisfactory sensitivity of 94.44% and a higher specificity (total specificity, 93.52%), especially in distinguishing non-neoplastic individuals, which displayed a specificity of 96.34%.

Despite the promising results, several limitations of this study should be acknowledged. First, the sample size, while sufficient for initial validation, remains relatively small and limited to a single cohort. This limitation may affect the generalizability of the findings, as the cohort may not fully capture the heterogeneity of UC across different populations and clinical settings. Second, the reliance on qMSP, while cost-effective and feasible, may limit the scalability of the assay in high-throughput clinical applications. Emerging technologies such as digital PCR or next-generation sequencing offer higher sensitivity, multiplexing capabilities, and scalability, which could be advantageous for broader clinical implementation. However, these technologies also come with higher costs and technical complexity, which may pose challenges for routine use in resource-limited settings. Future studies should explore the feasibility of integrating qMSP with these advanced technologies to balance cost-effectiveness and scalability. Third, while the three selected markers demonstrated excellent diagnostic performance, the biological mechanisms underlying their hypermethylation and its functional impact on UC progression remain incompletely understood. This gap in knowledge limits our ability to fully interpret the clinical significance of these markers and their potential role in UC pathogenesis.

In addition, we acknowledge several limitations related to the data and analytical methods used in this study. First, we utilized the Area Under the ROC Curve (AUC) as the primary metric for evaluating model performance, prioritizing models with higher AUC values. However, it must be recognized that AUC, as an assessment criterion, has inherent limitations. While AUC reflects the overall performance of models across all possible thresholds, it does not capture the variations in performance at specific thresholds. In clinical diagnostics, the choice of threshold can significantly impact the rates of false negatives and false positives, which are critical for clinical decision-making. Therefore, a high AUC does not necessarily guarantee that a model will meet clinical requirements in practice. To address this limitation, we selected a fixed threshold in this study to calculate specific sensitivity and specificity values. Additionally, we reported other metrics to complement AUC and provide a more comprehensive evaluation of the diagnostic model’s clinical applicability. Second, during the DMG discovery phase, we relied on the TCGA database to identify methylation differences between bladder cancer tissues and adjacent non-tumor tissues. However, the biomarkers identified in this phase were ultimately intended to distinguish cancer from non-cancer individuals. Previous studies have demonstrated that there can be significant epigenetic differences between adjacent non-tumor tissues and truly normal tissues [41, 42]. Due to ethical considerations, obtaining truly normal tissue samples is unproper, both in public databases and in clinical research. To address this limitation in future studies, we plan to use urine samples from healthy individuals to further validate and refine candidate biomarkers. This approach will help correct for potential biases and minimize the impact of this limitation when the final diagnostic model is applied in clinical practice.

Future research should involve larger-scale, multi-center studies to validate the efficacy of these findings in clinical application, referring to the clinical diagnostic biomarker study conducted by Zhang et al. [43], as well as Nie et al.’s study [18]. Additionally, comparative cost/benefit analyses, as well as integration with other liquid biopsy techniques, could further define the clinical utility of this approach in routine practice and optimize application scenarios to maximize patient benefit. By exploring the genomic and epigenetic landscapes of these markers in greater depth, future studies may elucidate the mechanistic connections between promoter methylation events and UC pathogenesis. Basic medical research, including the use of animal models and cell lines, could involve more molecular biology experiments to conduct comprehensive matrix studies, thereby enhancing the diagnostic model and advancing precision oncology.

## Conclusions

Our study demonstrates the potential of a three-marker diagnostic model (*VIM*, *TMEM220*, and *PPM1 N*) as a highly accurate and sensitive tool for the non-invasive detection of urothelial cancer. This model demonstrates robust performance, particularly in detecting UC at early stages, underscoring its promising clinical utility. These findings provide a strong foundation for future clinical applications and suggest that this diagnostic approach could improve early detection, guide treatment decisions, and ultimately enhance patient outcomes in urothelial cancer management.

## Supplementary Information

Additional file 1.

## Data Availability

The original contributions presented in this study are included in the article/supplementary material. Further inquiries can be directed to the corresponding authors.

## Abbreviations

utDNA: Urinary tumor DNA
WGBS: Whole genome bisulfite sequencing
UC: Urothelial carcinoma
BLCA: Bladder urothelial carcinoma
qMSP: Quantitative methylation-specific PCR
PPV: Positive predictive value
NPV: Negative predictive value

## References

[1] Ferlay J, Randi G, Bosetti C, Levi F, Negri E, Boyle P, La Vecchia C. Declining mortality from bladder cancer in Europe. BJU Int. 2008;101(1):11–9. 17971176 10.1111/j.1464-410X.2007.07239.x

[2] Sung H, Ferlay J, Siegel RL, Laversanne M, Soerjomataram I, Jemal A, Bray F. Global cancer statistics 2020: GLOBOCAN estimates of incidence and mortality worldwide for 36 cancers in 185 countries. CA: A Cancer J Clin. 2021;71(3):209–49. 10.3322/caac.21660 33538338

[3] Jubber I, Ong S, Bukavina L, Black PC, Compérat E, Kamat AM, Kiemeney L, Lawrentschuk N, Lerner SP, Meeks JJ, et al. Epidemiology of bladder cancer in 2023: a systematic review of risk factors. Eur Urol. 2023;84(2):176–90. 37198015 10.1016/j.eururo.2023.03.029

[4] Lefort F, Rhanine Y, Larroquette M, Domblides C, Heraudet L, Sionneau B, Lambert S, Lasserre M, Robert G, Ravaud A, et al. Clinical and biological differences between upper tract carcinoma and bladder urothelial cancer, including implications for clinical practice. Cancers. 2023;15(23):5558. 38067262 10.3390/cancers15235558 PMC10705302

[5] Saginala K, Barsouk A, Aluru JS, Rawla P, Padala SA, Barsouk A. Epidemiology of bladder cancer. Med Sci (Basel, Switzerland). 2020;8(1):15. 10.3390/medsci8010015 PMC7151633 32183076

[6] Catto JWF, Gordon K, Collinson M, Poad H, Twiddy M, Johnson M, Jain S, Chahal R, Simms M, Dooldeniya M, et al. Radical cystectomy against intravesical BCG for high-risk high-grade nonmuscle invasive bladder cancer: results from the randomized controlled BRAVO-feasibility study. J Clin Oncol: Off J Am Soc Clin Oncol. 2021;39(3):202–14. 10.1200/JCO.20.01665 PMC8078404 33332191

[7] Wu S, Li R, Jiang Y, Yu J, Zheng J, Li Z, Li M, Xin K, Wang Y, Xu Z, et al. Liquid biopsy in urothelial carcinoma: detection techniques and clinical applications. Biomed Pharmacother Biomed Pharmacother. 2023;165:115027. 37354812 10.1016/j.biopha.2023.115027

[8] Galceran J, Parada D, Eden M, Tumino R, Warren AY, Martos C, Neamtiu L, Visser O, Daubisse-Marliac L. The 2022 ENCR recommendations on recording and reporting of urothelial tumours of the urinary tract. Front Oncol. 2022;12:1046239. 36505871 10.3389/fonc.2022.1046239 PMC9727225

[9] Honkisz SI, Naughton JF, Weng HY, Fourez LM, Knapp DW. Evaluation of two-dimensional ultrasonography and computed tomography in the mapping and measuring of canine urinary bladder tumors. Veterinary J (London, England : 1997). 2018;232:23–6. 10.1016/j.tvjl.2017.12.008 29428087

[10] Boman H, Hedelin H, Holmng S. Four bladder tumor markers have a disappointingly low sensitivity for small size and low grade recurrence. J Urol. 2002;167(1):80–3. 11743280

[11] Mancini M, Righetto M, Zumerle S, Montopoli M, Zattoni F. The bladder EpiCheck test as a non-invasive tool based on the identification of DNA methylation in bladder cancer cells in the urine: a review of published evidence. Int J Mol Sci. 2020;21(18):6542. 32911596 10.3390/ijms21186542 PMC7554931

[12] Witjes JA, Morote J, Cornel EB, Gakis G, van Valenberg FJP, Lozano F, Sternberg IA, Willemsen E, Hegemann ML, Paitan Y, et al. Performance of the bladder EpiCheck? methylation test for patients under surveillance for non-muscle-invasive bladder cancer: results of a multicenter, prospective. Blind Clin Trial Eur Urol Oncol. 2018;1(4):307–13. 10.1016/j.euo.2018.06.011 31100252

[13] Liu C, Tang H, Hu N, Li T. Methylomics and cancer: the current state of methylation profiling and marker development for clinical care. Cancer Cell Int. 2023;23(1):242. 37840147 10.1186/s12935-023-03074-7 PMC10577916

[14] Xiao Y, Ju L, Qian K, Jin W, Wang G, Zhao Y, Jiang W, Liu N, Wu K, Peng M, et al. Non-invasive diagnosis and surveillance of bladder cancer with driver and passenger DNA methylation in a prospective cohort study. Clin Transl Med. 2022;12(8):e1008. 35968916 10.1002/ctm2.1008 PMC9377153

[15] Wang P, Shi Y, Zhang J, Shou J, Zhang M, Zou D, Liang Y, Li J, Tan Y, Zhang M, et al. UCseek: ultrasensitive early detection and recurrence monitoring of urothelial carcinoma by shallow-depth genome-wide bisulfite sequencing of urinary sediment DNA. EBioMedicine. 2023;89:104437. 36758479 10.1016/j.ebiom.2023.104437 PMC9941055

[16] Morris TJ, Butcher LM, Feber A, Teschendorff AE, Chakravarthy AR, Wojdacz TK, Beck S. ChAMP: 450k chip analysis methylation pipeline. Bioinform (Oxford, England). 2014;30(3):428–30. 10.1093/bioinformatics/btt684 PMC3904520 24336642

[17] Krueger F, Andrews SR. Bismark: a flexible aligner and methylation caller for Bisulfite-Seq applications. Bioinform (Oxford, England). 2011;27(11):1571–2. 10.1093/bioinformatics/btr167 PMC3102221 21493656

[18] Nie Y, Gao X, Cai X, Wu Z, Liang Q, Xu G, Liu N, Gao P, Deng J, Xu H, et al. Combining methylated SEPTIN9 and RNF180 plasma markers for diagnosis and early detection of gastric cancer. Cancer Commun (London, England). 2023;43(11):1275–9. 10.1002/cac2.12478 PMC10631480 37584087

[19] Zhang C, Xu X, Wang T, Lu Y, Lu Z, Wang T, Pan Z. Clinical performance and utility of a noninvasive urine-based methylation biomarker: TWIST1/Vimentin to detect urothelial carcinoma of the bladder. Sci Rep. 2024;14(1):7941. 38575639 10.1038/s41598-024-58586-7 PMC10995167

[20] Kulis M, Esteller M. DNA methylation and cancer. Adv Genet. 2010;70:27–56. 20920744 10.1016/B978-0-12-380866-0.60002-2

[21] Bennett RL, Licht JD. Targeting epigenetics in cancer. Annu Rev Pharmacol Toxicol. 2018;58:187–207. 28992434 10.1146/annurev-pharmtox-010716-105106 PMC5800772

[22] Klutstein M, Nejman D, Greenfield R, Cedar H. DNA methylation in cancer and aging. Can Res. 2016;76(12):3446–50. 10.1158/0008-5472.CAN-15-3278 27256564

[23] Wang W, Wang J, Li Z, Zhu M, Zhang Z, Wang Y, Jing H. Promoter hypermethylation of PTPL1, PTPN6, DAPK, p16 and 5-azacitidine inhibits growth in DLBCL. Oncol Rep. 2016;35(1):139–46. 26498513 10.3892/or.2015.4347

[24] Ridge KM, Eriksson JE, Pekny M, Goldman RD. Roles of vimentin in health and disease. Genes Dev. 2022;36(7–8):391–407. 35487686 10.1101/gad.349358.122 PMC9067405

[25] Jung S, Yi L, Kim J, Jeong D, Oh T, Kim CH, Kim CJ, Shin J, An S, Lee MS. The role of vimentin as a methylation biomarker for early diagnosis of cervical cancer. Mol Cells. 2011;31(5):405–11. 21491170 10.1007/s10059-011-0229-x PMC3887602

[26] Shirahata A, Hibi K. Serum vimentin methylation as a potential marker for colorectal cancer. Anticancer Res. 2014;34(8):4121–5. 25075038

[27] Shirahata A, Sakuraba K, Kitamura Y, Yokomizo K, Gotou T, Saitou M, Kigawa G, Nemoto H, Sanada Y, Hibi K. Detection of vimentin methylation in the serum of patients with gastric cancer. Anticancer Res. 2012;32(3):791–4. 22399595

[28] Satelli A, Li S. Vimentin in cancer and its potential as a molecular target for cancer therapy. Cell Mol Life Sci: CMLS. 2011;68(18):3033–46. 21637948 10.1007/s00018-011-0735-1 PMC3162105

[29] Choi B, Han TS, Min J, Hur K, Lee SM, Lee HJ, Kim YJ, Yang HK. MAL and TMEM220 are novel DNA methylation markers in human gastric cancer. Biomark: Biochem Indic Expo Response Susceptibility Chem. 2017;22(1):35–44. 10.1080/1354750X.2016.1201542 27329150

[30] Zhu L, Sun H, Tian G, Wang J, Zhou Q, Liu P, Tang X, Shi X, Yang L, Liu G. Development and validation of a risk prediction model and nomogram for colon adenocarcinoma based on methylation-driven genes. Aging. 2021;13(12):16600–19. 34182539 10.18632/aging.203179 PMC8266312

[31] Li T, Guan L, Tang G, He B, Huang L, Wang J, Li M, Bai Y, Li X, Zhang H. Downregulation of TMEM220 promotes tumor progression in hepatocellular carcinoma. Cancer Gene Ther. 2022;29(6):835–44. 34321624 10.1038/s41417-021-00370-0

[32] Gonzalez-Ramiro H, Parrilla I, Miquel Cambra J, Gonzalez-Plaza A, Antonia Gil M, Cuello C, Martinez EA, Rodriguez-Martinez H, Martinez CA. Combined synchronization and superovulation treatments negatively impact embryo viability possibly by the downregulation of WNT/β-catenin and notch signaling genes in the porcine endometrium. J Anim Sci. 2022. 10.1093/jas/skac315. 36169657 10.1093/jas/skac315 PMC9683510

[33] Kamada R, Kudoh F, Ito S, Tani I, Janairo JIB, Omichinski JG, Sakaguchi K. Metal-dependent Ser/Thr protein phosphatase PPM family: evolution, structures, diseases and inhibitors. Pharmacol Ther. 2020;215:107622. 32650009 10.1016/j.pharmthera.2020.107622

[34] Babjuk M, Burger M, Capoun O, Cohen D, Compérat EM, Dominguez Escrig JL, Gontero P, Liedberg F, Masson-Lecomte A, Mostafid AH, et al. European association of urology guidelines on non-muscle-invasive bladder cancer (Ta, T1, and carcinoma in situ). Eur Urol. 2022;81(1):75–94. 34511303 10.1016/j.eururo.2021.08.010

[35] Dimashkieh H, Wolff DJ, Smith TM, Houser PM, Nietert PJ, Yang J. Evaluation of urovysion and cytology for bladder cancer detection: a study of 1835 paired urine samples with clinical and histologic correlation. Cancer Cytopathol. 2013;121(10):591–7. 23801650 10.1002/cncy.21327 PMC3800248

[36] Lin T, Liu Z, Liu L, Yang L, Han P, Zhang P, Wei Q. Prospective evaluation of fluorescence in situ hybridization for diagnosing urothelial carcinoma. Oncol Lett. 2017;13(5):3928–34. 28529600 10.3892/ol.2017.5926 PMC5431676

[37] Koenig F, Jung K, Schnorr D, Loening SA. Urinary markers of malignancy. Clin Chimica Acta Int J Clin Chem. 2000;297:191–205. 10.1016/s0009-8981(00)00246-1 10841921

[38] Rink M, Chun FK, Dahlem R, Soave A, Minner S, Hansen J, Stoupiec M, Coith C, Kluth LA, Ahyai SA, et al. Prognostic role and HER2 expression of circulating tumor cells in peripheral blood of patients prior to radical cystectomy: a prospective study. Eur Urol. 2012;61(4):810–7. 22277196 10.1016/j.eururo.2012.01.017

[39] Têtu B. Diagnosis of urothelial carcinoma from urine. Modern Pathol : An Off J United States and Can Acad Pathol. 2009;22:S53-9. 10.1038/modpathol.2008.193 19494853

[40] Chen X, Zhang J, Ruan W, Huang M, Wang C, Wang H, Jiang Z, Wang S, Liu Z, Liu C, et al. Urine DNA methylation assay enables early detection and recurrence monitoring for bladder cancer. J Clin Invest. 2020;130(12):6278–89. 32817589 10.1172/JCI139597 PMC7685755

[41] Spitzwieser M, Entfellner E, Werner B, Pulverer W, Pfeiler G, Hacker S, Cichna-Markl M. Hypermethylation of CDKN2A exon 2 in tumor, tumor-adjacent and tumor-distant tissues from breast cancer patients. BMC Cancer. 2017;17(1):260. 28403857 10.1186/s12885-017-3244-2 PMC5389179

[42] Widayati TA, Schneider J, Panteleeva K, Chernysheva E, Hrbkova N, Beck S, Voloshin V, Chervova O. Open access-enabled evaluation of epigenetic age acceleration in colorectal cancer and development of a classifier with diagnostic potential. Front Genet. 2023;14:1258648. 37953923 10.3389/fgene.2023.1258648 PMC10634722

[43] Zhang R, Nie Y, Chen X, Jiang T, Wang J, Peng Y, Zhou G, Li Y, Zhao L, Chen B, et al. A multicenter prospective clinical trial reveals cell free DNA methylation markers for early esophageal cancer. J Clin Invest. 2025. 10.1172/JCI186816. 39998886 10.1172/JCI186816 PMC11996849

