# Supplementary material for: A diagnostic model for non-invasive urothelial cancer early detection based on methylation of urinary tumor DNA
Source: Cancer Cell Int. 2025 Apr 15;25:148. doi: 10.1186/s12935-025-03766-2 (PMC12001437; doi:10.1186/s12935-025-03766-2)
Supplement: Supplementary file 1 — Additional file 1. [file 12935_2025_3766_MOESM1_ESM.docx]

**Supplemental materials**

**Supplemental Figures**

**
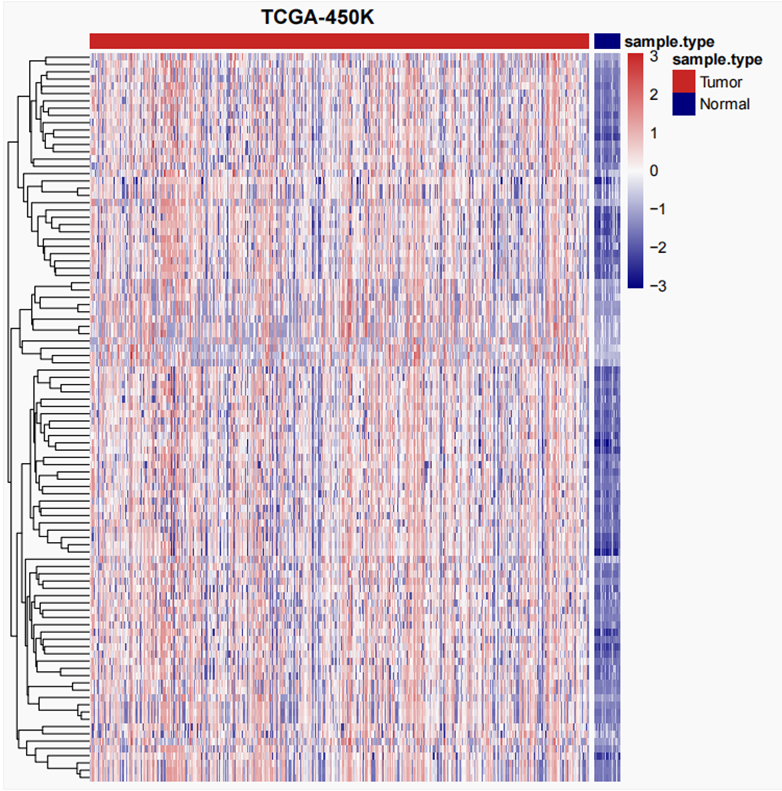
**

**Supplemental Figure 1.** The heatmap displaying differential methylation genome sites hypermethylated in bladder cancer tissues relative to normal tissues. The data was from TCGA BLCA datasets based on WGBS 450K array. The scale bars indicate the methylation levels scaled by Z-score. Red on scale bar means high level and blue means low level.


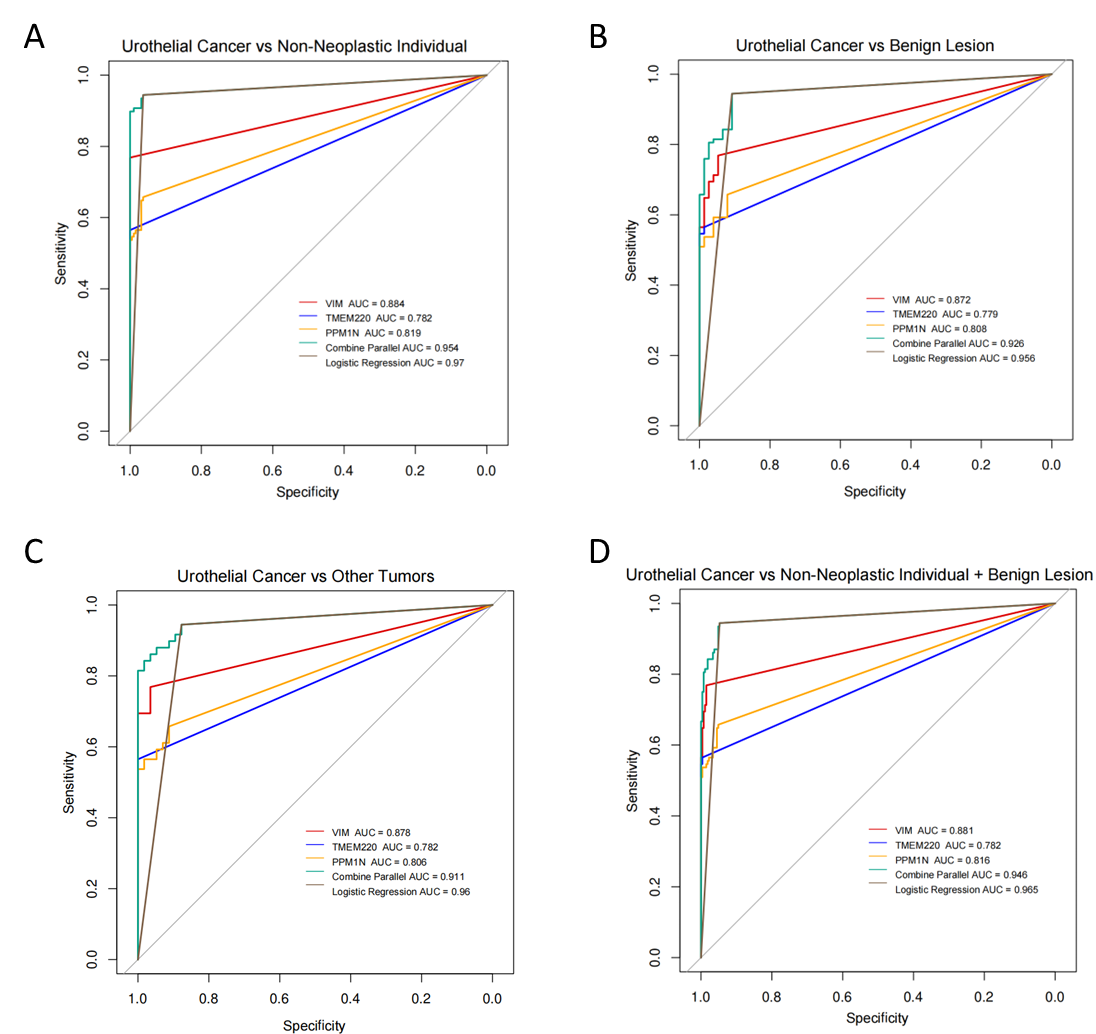


**Supplemental Figure 2. Performance of the three-gene diagnostic model in the model-testing cohort. (A-D)** The ROC curves of three single methylation DNA markers, Combine Parallel (three-gene diagnostic model) and logistic regression model in the model-testing cohort for distinguishing UC patients from other kinds of cases, including UC patients vs. non-neoplastic individuals **(A)**, UC patients vs. benign lesions patients **(B)**, UC patients vs. other cancers patients **(C)**, UC patients vs. non-neoplastic cases and benign lesions patients **(D)**. The AUC values for the different diagnostic markers were marked in the graph legend of each plot. Three-gene diagnostic model was based on the logic of single-gene positive indicating positive and triple negative indicating negative. Logistic regression model was based on the logistic regression algorithm of *VIM*, *TMEM220* and *PPM1N*.

**
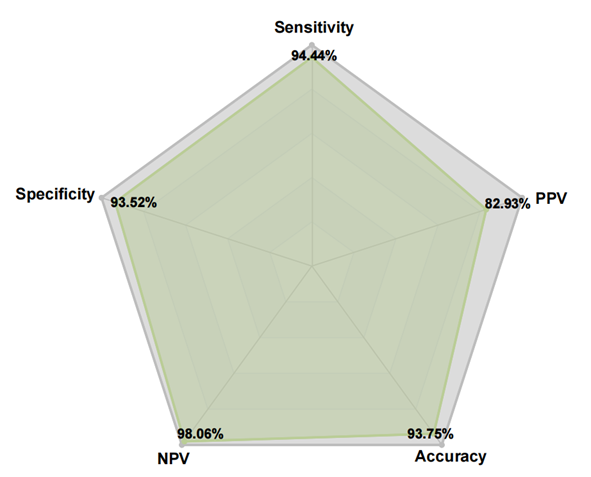
**

**Supplemental Figure 3. Radar chart presented the model performance.** Radar chart displayed 5 kinds of performance indicators. NPV, Negative Predictive Value. PPV, Positive Predictive Value.
